# Unique Gene Expression Patterns in Human T-cell Lines Generated from Multiple Sclerosis Patients by Stimulation with a Synthetic MOG Peptide

**DOI:** 10.1080/17402520500233460

**Published:** 2005-09

**Authors:** Mathilda Mandel, Michael Gurevich, Gad Lavie, Irun R. Cohen, Anat Achiron

**Affiliations:** 1 Blood Center Tel-Aviv University. -aviv Israel; 2 Sackler School of Medicine Tel-Aviv University Tel-Aviv Israel; 3 Multiple Sclerosis Center Tel-Aviv University Tel-Aviv Israel; 4 Functional Genomics Unit Sheba Medical Center Tel-Hashomer Tel-Aviv University Tel-Aviv Israel; 5 Department of Immunology Weizmann Institute of Science Rehovot Tel-Aviv University Tel-Aviv Israel

## Abstract

Multiple sclerosis (MS) is an autoimmune disease where T-cells activated against myelin antigens are involved in myelin destruction. Yet, healthy subjects also harbor T-cells responsive to myelin antigens, suggesting that MS patient-derived autoimmune T-cells might bear functional differences from T-cells derived from healthy individuals. We addressed this issue by analyzing gene expression patterns of myelin oligodendrocytic glycoprotein (MOG) responsive T-cell lines generated from MS patients and healthy subjects. We identified 150 transcripts that were differentially expressed between MS patients and healthy controls. The most informative 43 genes exhibited >1.5-fold change in expression level. Eighteen genes were up-regulated including BCL2, lifeguard, IGFBP3 and VEGF. Twenty five genes were down-regulated, including apoptotic activators like TNF and heat shock protein genes. This gene expression pattern was unique to MOG specific T-cell lines and was not expressed in T-cell lines reactive to tetanus toxin (TTX). Our results indicate that activation in MS that promotes T-cell survival and expansion, has its own state and that the unique gene expression pattern that characterize autoreactive T-cells in MS represent a constellation of factors in which the chronicity, timing and accumulation of damage make the difference between health and disease.


*Clinical & Developmental Immunology*, September 2005; 12(3): 203–209

Taylor & Francis

Taylor & Francis Group

### Unique gene expression patterns in human T-cell lines generated from multiple sclerosis patients by stimulation with a synthetic MOG peptide

MATHILDA MANDEL[1,2], MICHAEL GUREVICH[3,4], GAD LAVIE[1], IRUN R. COHEN[5], & ANAT ACHIRON[2,3]

[1]*Blood Center, Tel-Aviv University, Tel-Aviv, Israel,* [2]*Sackler School of Medicine, Tel-Aviv University, Tel-Aviv, Israel,* [3]*Multiple Sclerosis Center, Tel-Aviv University, Tel-Aviv, Israel,* [4]*Functional Genomics Unit, Sheba Medical Center, Tel-Hashomer, Tel-Aviv University, Tel-Aviv, Israel, and* [5]*Department of Immunology, Weizmann Institute of Science, Rehovot Tel-Aviv University, Tel-Aviv, Israel*

**Abstract**
Multiple sclerosis (MS) is an autoimmune disease where T-cells activated against myelin antigens are involved in myelin destruction. Yet, healthy subjects also harbor T-cells responsive to myelin antigens, suggesting that MS patient-derived autoimmune T-cells might bear functional differences from T-cells derived from healthy individuals. We addressed this issue by analyzing gene expression patterns of myelin oligodendrocytic glycoprotein (MOG) responsive T-cell lines generated from MS patients and healthy subjects. We identified 150 transcripts that were differentially expressed between MS patients and healthy controls. The most informative 43 genes exhibited $>1.5$-fold change in expression level. Eighteen genes were up-regulated including BCL2, lifeguard, IGFBP3 and VEGF. Twenty five genes were down-regulated, including apoptotic activators like TNF and heat shock protein genes. This gene expression pattern was unique to MOG specific T-cell lines and was not expressed in T-cell lines reactive to tetanus toxin (TTX). Our results indicate that activation in MS that promotes T-cell survival and expansion, has its own state and that the unique gene expression pattern that characterize autoreactive T-cells in MS represent a constellation of factors in which the chronicity, timing and accumulation of damage make the difference between health and disease.

**Keywords:** *Autoimmunity, gene expression, multiple sclerosis, T-cell lines*

### Introduction

Multiple sclerosis (MS) is a chronic inflammatory demyelinating disease of the central nervous system and the most common cause of disability in young adults. The etiology of MS involves autoimmune mechanisms within the background of genetic susceptibility (Compston and Sawcer 2002). Myelin sheath-derived antigenic epitopes involving predominantly myelin basic protein (MBP), myelin oligodendrocyte glycoprotein (MOG) and the proteolipid protein (PLP) are targets for the autoimmune T-cell activation in MS patients. These autoreactive T-cells cause inflammatory reaction leading to myelin damage and axonal loss (Stinissen et al. 1998, Gran and Rostami 2001). The autoreactive T-cells can be generated and propagated as specific T-cell lines from MS patients and are being used following attenuation to vaccinate MS patients against the disease (Zhang et al. 1993, Achiron et al. 2004). The frequency of autoimmune T-cells is influenced by immune regulatory mechanisms involving cytokine stimulation and apoptotic deletion (Pender 1998). It has been reported that activated T-cell lines responsive to MBP, MOG and other myelin epitopes can be also propagated from peripheral blood mononuclear cells of healthy subjects (Ota et al. 1990). This fact led us to examine whether autoreactive T-cell lines derived from MS patients bear functional differences relative to T-cell lines obtained from healthy subjects, in order to evaluate whether these differences bear implications for the

Correspondence: M. Mandel, Blood Center, Sheba Medical Center, Tel Hashomer 52621, Israel. Tel: 972 3 5304532. Fax: 972 3 5303072. E-mail: mandel@sheba.health.gov.il

ISSN 1740-2522 print/ISSN 1740-2530 online © 2005 Taylor & Francis
DOI: 10.1080/17402520500233460

pathogenesis and regulation of MS. We applied microarray analysis (Duggan et al. 1999) to determine gene expression of MOG stimulated T-cell lines derived from MS patients in comparison to healthy controls. We chose to study T-cells responsive to MOG as this myelin epitope is expressed preferentially on the outermost surface of the myelin sheath, is highly immunogenic and was reported to induce severe experimental autoimmune encephalomyelitis (EAE) in both rodents and primates (Zhong et al. 2000), as well as to serve a major target for autoimmune T-cells in MS patients (Weissert et al. 2002).

### Materials and methods

*Subjects*

Four female patients with definite MS and a relapsing-remitting course (mean age $38 + 4.2$ years, mean disease duration $9.3 + 3.3$ years) and three age- and sex-matched healthy subjects were included in the study. None of the patients received immunomodulatory drugs or steroid treatment for at least 3 months prior to blood drawn. The study was approved by the institutional review board and the Israel Ministry of Health. Written informed consent was obtained from all participants.

*Generation of MOG-reactive T-cell lines*

Peripheral blood mononuclear cells (PBMC) were separated on ficoll-hypaque and plated in 96-well U-bottom microplates in the presence of MOG peptide (aa residues 34–56, 15 μg/ml, Chiron) as previously described (Zhang et al. 1993). Tetanus toxin (TTX) peptide (aa 176–195, 15 μg/ml, Hy-labs, Israel) was used as a non-encephalitogenic control antigen. Selection of wells for expansion of lines was performed by $^{3}$H-thymidine incorporation into subdivided primary cultures propagated with the peptide antigens for 7 days and yielding a stimulation index $>3$ relative to control cells grown without the peptide. The wells were examined for IFN-γ and IL-10 production by RT-PCR. Wells positive for IFN-γ and low or negative for IL-10 were selected (Th1 cells). These cells were subcultured on freshly obtained antigen presenting cells (irradiated PBMC) and antigen reactive T-cell lines were generated following expansion with IL-2, 50 U/ml (Boehringer Mannheim, Germany). Cells for analysis from both MS patients and control donors were harvested for experiments 14–21 days after line selection and expansion over IL-2 at the peak of the logarithmic growth phase. T-cell lines were examined for CD4/CD8 ratio and those with CD4 content $>60\%$ were used for gene microarray analyses.

*mRNA preparation*

Total RNA was isolated from MOG stimulated T-cell lines ($2 \times 10^{7}$ cells) by ice-cold TRIZOL Reagent (Gibco BRL). Poly-A mRNA was isolated with mini-kit (Oligotex, Qiagen) and used as a template for double stranded cDNA synthesis oligo (dT)-24 primers containing a T7 RNA polymerase promoter site added to the 3′- end (Genset). After phenol/chloroform extraction cDNA was used as a template for *in-vitro* transcription (Ambion T7 Megascript system) with biotin labeled nucleotides (Enzo Diagnostics). Labeled cRNA was fragmented, quantified by spectrophotometer, and hybridized.

*Microarray gene analysis*

Each Genechip (U95Av2, 12,000 genes) was hybridized with 10 μg/200 μl hybridization mix, stained and scanned (Hewlett Packard, GeneArray-TM scanner G2500A) according to manufacturer protocol (Affymetrix Inc, Santa Clara, CA). MAS5 software (Affymetrix Inc.) was used to analyze the scanned arrays. All data were normalized by dChip software. Probes that did not have an expression value of 100 in at least one of the arrays were filtered.

*Data analysis*

The analysis was performed according to the analytical approach as previously described (Lovett-Racke et al. 1998, Ben-Dor et al. 2000, Ria et al. 2001). Fold ratios were calculated for each gene of the samples against the geometric mean of matched controls. To determine the most informative genes threshold number of misclassifications (TNoM) score was applied (Rechsteiner et al. 2000). This score counts the number of classification errors that occur between compared groups for each gene of the dataset. The best threshold ($TNoM = 0$) implies that no errors have been counted and the distinction between the two groups in relation to the expression level of a specific gene is maximal. To select a group of genes with strongly differential expression, we also computed $t$-test $p$-value (comparing expression levels of genes from MS patients vs. healthy controls). Genes with $TNoM = 0$, fold-change $>1.5$ (either up or down regulated) and corresponding $t$-test $p$ value $<0.05$, were designated as most informative.

*Confirmation of microarray gene expression*

Real-Time RT-PCR analysis was preformed for three most informative genes using the QuantiTect SYBER Green PCR kit (QIAGEN) and specific primers for IGF-BP3 (5′- AGAATATGGTCCCTGCCGTAGA-3′, 5′- TTCAGGTGATTCAGTGTGTCTTCC-3′),

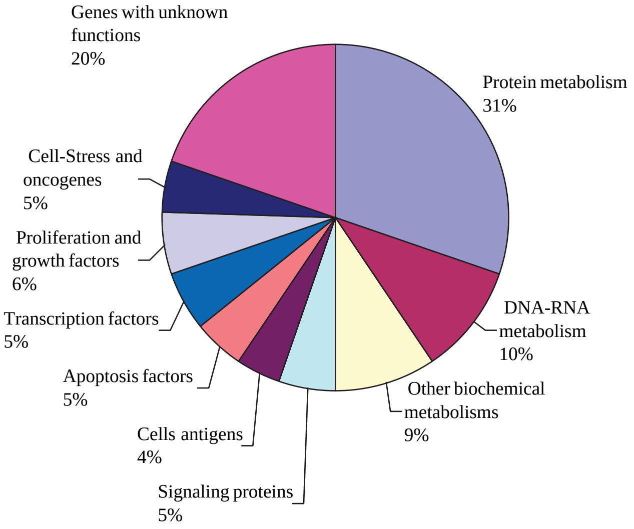

Figure 1. Pie-chart diagram showing the functional groups of the 150 differentially expressed gene transcripts.

VEGF (5′ACACAGACTCGCGTTGCAAG-3′, 5′—CACATCTGCAAGTACGTTCGTTTA-3′) and BLVRA (5′- ACCTCAGTCCCACGACAGT-TATG-3′, 5′—AGGAAAGCCGAGGTGCAG-3′). β-Actin (5′- CCTGGCACCCAGCACAAT-3′, 5′-GCCGATCCACACGGAGTACT-3′) was used as a control. Samples were run in triplicates and average fold change calculated.

### Results

Gene expression patterns obtained from MOG reactive T-cell lines from MS patients disclosed 150 differentially expressed transcripts with TNoM = 0, as compared to healthy subjects. A pie-chart diagram showing the functional groups of these genes is presented in Figure 1.

Hierarchical clustering of the 150 differentially expressed genes between MS patients and healthy controls is presented in Figure 2A. We further identified and clustered 43 most informative genes, 18 up-regulated and 25 down-regulated genes (Figure 2B).

Analysis of the known biological function of these genes demonstrated an apparently diverse portfolio of activities, Table I. We identified genes coding for proteins involved in the regulation and execution of apoptosis, growth factors, mediators of signal transduction pathways, molecules that participate in inflammation and also genes encoding heat shock proteins, transcription factors and components of different biochemical pathways. More specifically, genes that were up-regulated in the MS patients-derived T-cell lines include growth factors such as vascular endothelial growth factor (VEGF), insulin-like growth factor binding protein 3 (IGF-BP3),

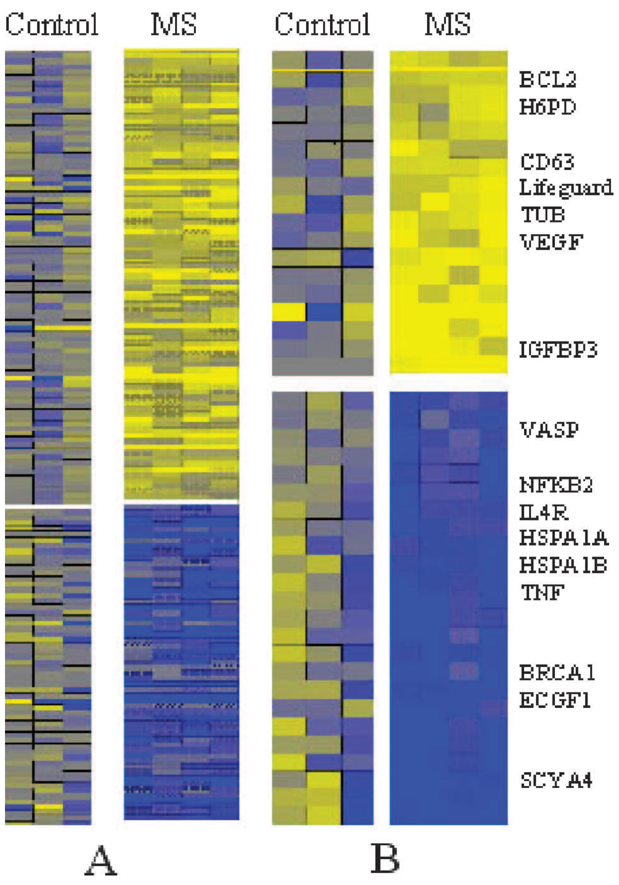

Figure 2. Infogram of gene expression levels of MOG stimulated T-cell lines from MS patients and healthy controls. (A) One hundred and fifty differentially expressed genes (TNoM = 0, $p < 0.05$). (B) Forty three most informative genes with TNoM = 0, fold change $>1.5$ and $p < 0.05$. Each row represents a gene and each column refers to a T-cell line from a different subject. Genes with increased expression (relative to average of control samples) are depicted in yellow, and genes with decreased expression are depicted in blue.

Table I. Functional characteristic of the most informative genes differentially expressed in MOG activated T-Cells between MS patients and healthy subjects.

| Identifier | Symbol | Name | Function | Fold Change | *P* value *t*-test |
|---|---|---|---|---|---|
| Up regulated | | | | | |
| M35878 | IGFBP3 | Insulin-like growth factor binding protein 3 | Modulates IGF activity | 5.8 | 0.03 |
| AB002318 | KIAA0320 | KIAA0320 protein | | 2.4 | 0.05 |
| AF024710 | VEGF | Vascular endothelial growth factor | Endothelial cell proliferation | 2.3 | 0.02 |
| AA628946 | KHSRP | KH-type splicing regulatory protein | mRNA processing | 2.2 | 0.01 |
| L42374 | PPP2R5B | Protein phosphatase 2, regulatory subunit B | Protein phosphatase | 2.1 | 0.05 |
| U54644 | TUB | Tubby (mouse) homolog | May be a transcription factor | 1.8 | 0.01 |
| AB023167 | KIAA0950 | Lifeguard (LFG) | Apoptosis | 1.8 | 0.006 |
| X62654 | CD63 | CD63 antigen (melanoma 1 antigen) | Growth regulation | 1.8 | 0.03 |
| H98552 | | cDNA DKFZp586I0523 | | 1.8 | 0.01 |
| AL050395 | MOF | Member of MYST acetyl transferases | Histone acetyl transferases | 1.7 | 0.03 |
| L27213 | SLC4A3 | Solute carrier family 4, anion exchange 3 | Inorganic anion exchanger | 1.7 | 0.01 |
| AF014837 | M6A | Putative methyltransferase | Transcription factor | 1.6 | 0.05 |
| AB014537 | KIAA0637 | KIAA0637 gene product | Apoptosis | 1.5 | 0.003 |
| D13969 | ZNF144 | Zinc finger protein 144 (Mel-18) | DNA-Binding protein | 1.5 | 0.04 |
| AJ012590 | H6PD | Hexose-6-phosphate dehydrogenase | Oxidoreductase | 1.5 | 0.04 |
| M13995 | BCL2 | B-cell CLL/lymphoma 2 | Apoptosis | 1.5 | 0.03 |
| AI760801 | | Chromosome 19, cosmid R31180 | | 1.5 | 0.009 |
| AI660963 | MAP3K12 | Mitogen-activated protein 3 kinase 12 | Transferase Cytoplasmic | 1.5 | 0.02 |
| Down regulated | | | | | |
| D45248 | PSME2 | Proteasome activator subunit 2 (PA28 β) | Protein degradation | − 1.5 | 0.04 |
| W28612 | | ESTs | | − 1.5 | 0.02 |
| Z46389 | VASP | Vasodilator-stimulated phosphoprotein | Signal transduction | − 1.6 | 0.02 |
| AA152202 | FLJ14639 | Hypothetical protein FLJ14639 | | − 1.6 | 0.02 |
| AF080561 | RBM14 | RNA binding motif protein 14 | RNA binding protein | − 1.7 | 0.03 |
| D50922 | KIAA0132 | Kelch-like ECH-associated protein 1 | ECH-associated protein 1 | − 1.7 | 0.03 |
| AF025441 | OIP5 | Opa-interacting protein 5 | | − 1.8 | 0.04 |
| AF080227 | EED | Embryonic ectoderm development | Transcriptional repressor | − 1.8 | 0.04 |
| D87957 | RQCD1 | Required for cell differentiation | Sex differentiation | − 1.9 | 0.03 |
| X61498 | NFKB2 | Nuclear factor of kappa light polypeptide Bcells | Expression of inflammatory genes | − 1.9 | 0.05 |
| X52425 | IL4R | Interleukin 4 receptor | Receptor signalling protein | − 2 | 0.04 |
| L08069 | DNAJA1 | DnaJ (Hsp40) homolog, subfamily A, member 1 | Protein folding and transport | − 2 | 0.04 |
| AF071504 | STX11 | Syntaxin 11 | Protein transport | − 2.1 | 0.03 |
| M11717 | HSPA1A | Heat shock 70 kDa protein 1A | Heat shock response | − 2.2 | 0.03 |
| M59830 | HSPA1B | Heat shock 70kD protein 1B | Heat shock response | − 2.2 | 0.03 |
| M16441 | TNF | Human tumor necrosis factor | Inflamatory response | − 2.3 | 0.05 |
| D89077 | SLA | Src-like-adapter | | − 2.4 | 0.05 |
| U77949 | CDC6 | Cell division cycle 6, S. cerevisiae homolog | DNA replication checkpoint | − 2.5 | 0.02 |
| D38549 | KIAA0068 | KIAA0068 protein | | − 2.5 | 0.01 |
| L23959 | TFDP1 | Transcription factor Dp-1 | Cycle progression G1 to S-phase | − 2.5 | 0.01 |
| L78833 | BRCA1 | Breast cancer susceptibility gene | | − 2.7 | 0.04 |
| M63193 | ECGF1 | Endothelial cell growth factor 1 | Stimulates angiogenesis | − 2.8 | 0.01 |
| AF035625 | STK11 | Serine/threonine kinase 11 | Peutz–Jeghers syndrome | − 2.9 | 0.04 |
| J04130 | SCYA4 | Small inducible cytokine A4 | Cell-to-cell signalling | − 2.9 | 0.05 |
| X93086 | BLVRA | Biliverdin reductase A | Biliverdin reductase | − 4 | 0.03 |

Table II. Quantitative real time RT-PCR analyses for three most informative genes from MOG responsive T-cell lines derived from MS patients relative to healthy subjects.

| Average fold change | RT PCR | Gene expression | Correlation coefficient |
|---|---|---|---|
| IGF-BP3 | 6.261 | 5.8 | 0.96 |
| VEGF | 2.3 | 2.3 | 1.0 |
| BLVRA | 0.704 | 0.55 | 0.72 |

CD63 melanoma-1 antigen which is also known to act as a growth regulator. Additionally anti-apoptotic genes such as B-cell CCL/Lymphoma 2 (BCL2) which blocks apoptosis by interfering with activation of caspases, and the lifeguard gene (LFG)—an anti-apoptotic component involved in inhibition of induction of apoptosis downstream to the activating signal generated by CD95/Fas receptor interaction with its ligand, and the transcript for MAP activated kinase were recognized. Down-regulated genes included the proteasome activator subunit 2 involved in the presentation of endogenous antigens by MHC class I molecules and required for efficient antigenic epitope processing (Chen et al. 2001), the 70 kDa heat shock proteins 1A and B (HSP-A1A, HSP-A1B), endothelial cell growth factor 1 (ECGF1), inflammatory cytokines such as small inducible cytokine A4 (SCYA4) which is involved in cell-to-cell signaling, tumor necrosis factor (TNF), interleukin-4 receptor (IL-4R) and nuclear factor of kappa light polypeptide gene enhancer B-cells (NF-KB2) which binds to the kappa-B consensus sequence located in the enhancer region of genes involved in immune response (Zettl et al. 1998). Real time RT-PCR of three of the most informative genes confirmed the microarray findings with high correlation between methods, Table II.

MOG responsive T-cell line gene expression signature was evaluated for specificity by comparison with T-cell lines stimulated by TTX as a non-encephalitogenic antigen. This comparison disclosed that only six of the 150 MOG stimulated differentially expressed genes (CLPTM1, VASP, PDAP1, NFKB2, SLA and BCL2), were common with the expression pattern of TTX stimulated lines.

### Discussion

In the present study, we demonstrated that MOG responsive T-cell lines derived from patients with MS have inherent gene transcriptional fingerprint. This transcriptional fingerprint differs from that observed in MOG activated T-cell lines generated in healthy subjects, although both responded to the same myelin antigen. Moreover, this gene expression pattern was found to be unique in MS as it was confined to MOG specific T-cell lines and not to T-cell lines reactive with a non-encephalitogenic TTX antigen. This suggests that the MOG stimulated expression signature in MS is indeed specific and relates to the encephalitogenic antigen.

The differentiating gene transcripts within the signature indicate a functional platform of activities that may be associated with disease pathogenesis. Up-regulated genes include anti-apoptotic genes such as BCL2, LFG and MAP3K12. The BCL2 gene product is an important member of the anti-apoptotic proteins. High numbers of BCL2 positive T lymphocytes were found in demyelinating and remyelinating brain lesions from MS patients (Zettl et al. 1998). Likewise, an increased frequency of MBP-reactive T-cells in MS patients relative to healthy individuals was detected when an antibody to Fas-ligand was used to block apoptosis (Zang et al. 1999). LFG is known to inhibit T-cell death mediated by the Fas receptor (CD95) through a unique mechanism that down regulates apoptotic signals from Fas (Somia et al. 1999). Loss of Fas/Fas ligand activities was reported in human autoimmune lymphoproliferative syndrome (Van den Berg et al. 2002), and in lymphoproliferative lupus-like syndrome in mice (Watanabe et al. 1992). The MAP3K12 gene is associated with programmed cell death and was reported to catalyze the phosphorylation of BAD, a member of the BCL2 protein family which stimulates BCL2 dependent anti-apoptotic pathways (Yang et al. 1995, Bonni et al. 1999). Another aspect of failure to induce apoptosis in the MS-derived T-cell lines is the significant down-regulation of the TNF pro-apoptotic gene. The inadequate apoptosis present in MS autoreactive T-cell lines could lead to insufficient deletion of autoimmune activated T-cell clones and increase susceptibility to autoimmunity. TNF is also known to stimulate MHC class I presentation in addition to induction of apoptosis. Our finding of down-regulation of MHC class I presentation include the PA28β involved in the presentation of endogenous antigens and required for efficient antigenic epitope processing. Accordingly, we suggest that a weaker antigenic MHC class I presenting capability might distinguish MS-patient derived T-cell lines from their healthy counterparts. It is conceivable that a lower expression of MHC class-I on CD4 autoimmune T-cells might enable them to escape regulation by CD8 cells that recognize autoimmune idiotypes (Zang et al. 2000). We also found higher expression of IGFBP3 and VEGF in MOG responsive T- cell lines derived from MS patients. IGFBP3 has previously been reported to be expressed on human stimulated and unstimulated lymphocytes (Kooijman et al. 1995). In murine EAE, IGF-1/IGFBP3 treatment resulted in more severe disease due to enhanced expansion of encephalitogenic T-cells (Lovett-Racke et al. 1998).

Our findings are in agreement with these observations, as well as with findings by others (Lock et al. 2002) that reported increased expression of IGFBP3 in acute brain lesions of MS patients compared to silent MS lesions. In addition to increased survival potential, our findings suggest that autoreactive T-cells in MS also have an expansion advantage compared with T-cells from healthy individuals. Furthermore, migration of autoimmune T-cells into the brain would be expected to be assisted by over-expression of VEGF. VEGF enhances vascular permeability and may facilitate migration of lymphocytes into the brain and induction of autoreactive inflammatory response. Taken together the combined effects of down-regulation of apoptosis associated genes, up regulation of antiapoptotic genes, increased expansion capability of autoreactive T-cells and enhanced ability to penetrate the brain may lead to perpetuated pathologic cellular proliferation and tissue destruction within the central nervous system, along with increased resistance to regulation. These specific gene transcripts can define some of the requirements for an individual to develop MS. It is of noteworthy that despite activation *in-vitro* with the same MOG epitope, MOG reactive T- cell lines from healthy subjects did not attain the gene expression profile that characterized the MS patient-derived cells. Thus, these findings support the concept that not all autoimmune T-cells are equal; autoimmune T-cells from MS patients follow a unique pattern of T-cell activation that appears to be more resilient to apoptosis and can support long term survival. Although, T-cell lines derived from MS patients and healthy donors responded to the same autoantigen, the gene expression imprints that are unique to each group were preserved. The nature of the stimuli that generate aberrant autoimmune T-cell gene expression has yet to be identified in order to determine whether their formation is merely the result of the chronic immune stimulation driven by other factors in MS, or whether such T-cells function are the primary drivers of the pathogenic MS process (Ria et al. 2001). On the other hand, the difference we demonstrated between autoreactive T-cells in patients and healthy subjects suggests that though both are activated and against the same myelin antigen their state is different, and the change in state is of importance. This transformation between autoimmune reactivity and autoimmune disease and the characterization of such driver autoreactive T-cells dictating the state of immunity–autoimmunity, may contribute to understanding autoimmune diseases and possibly contribute to the designation of effective treatments in MS.

### References

Achiron A, Lavie G, Kishner I, Stern Y, Sarova-Pinhas I, Ben-Aharon T, Barak Y, Raz H, Lavie M, Barliya T, Faibel M, Cohen IR, Mandel M. 2004. T-cell vaccination in multiple sclerosis relapsing–remitting nonresponders patients. Clin Immunol 113:155–160.

Ben-Dor A, Bruhn L, Fridman N, Nachman I, Schummer M, Yakhini Z. 2000. Tissue classification with gene expression profiles. J Comput Biol 7:559–583.

Bonni A, Brunet A, West AE, Datta SR, Takasu MA, Greenberg ME. 1999. Cell survival promoted by the Ras-MAPK signaling pathway by transcription-dependent and -independent mechanisms. Science 286:1358.

Chen F, Castranova V, Shi X. 2001. New insights into the role of nuclear factor-kappaB in cell growth regulation. Am J Pathol 159:387–397.

Compston A, Sawcer S. 2002. Genetic analysis of multiple sclerosis. Curr Neurol Neurosci Rep 2:259–266.

Duggan DJ, Bittner M, Chen Y, Meltzer P, Trent JM. 1999. Expression profiling using cDNA microarrays. Nat Genet 21:10–14.

Gran B, Rostami A. 2001. T-cells, cytokines, and autoantigens in multiple sclerosis. Curr Neurol Neurosci Rep 1:263–270.

Kooijman R, Scholtens LE, Rijkers GT, Zegers BJ. 1995. Type I insulin-like growth factor receptor expression in different developmental stages of human thymocytes. J Endocrinol 147:203–209.

Lock C, Hermans G, Pedotti R, Brendolan A, Schadt E, Garren H, Langer G, Strober S, Cannella B, Allard J, Klonowski P, Austin A, Lad N, Kaminski N, Galli SJ, Oksenberg JR, Raine CS, Heller R, Steinman L. 2002. Gene-microarray analysis of multiple sclerosis lesions yields new targets validated in autoimmune encephalomyelitis. Nat Med 8:500–508.

Lovett-Racke AE, Bittner P, Cross AH, Carlino JA, Racke MK. 1998. Regulation of experimental autoimmune encephalomyelitis with insulin-like growth factor (IGF-1) and IGF-1/IGF-binding protein-3 complex (IGF-1/IGFBP3). J Clin Investig 101:1797–1804.

Ota K, Matsui M, Milford EL, Mackin GA, Weiner HL, Hafler DA. 1990. T-cell recognition of an immunodominant myelin basic protein epitope in multiple sclerosis. Nature 346:183–187.

Pender MP. 1998. Genetically determined failure of activation-induced apoptosis of autoreactive T-cells as a cause of multiple sclerosis. Lancet 351:978–981.

Rechsteiner M, Realini C, Ustrell V. 2000. The proteasome activator 11 S REG (PA28) and class I antigen presentation. Biochem J 345(Pt 1):1–15.

Ria F, van den Elzen P, Madakamutil LT, Miller JE, Maverakis E, Sercarz EE. 2001. Molecular characterization of the T-cell repertoire using immunoscope analysis and its possible implementation in clinical practice. Curr Mol Med 3:297–304.

Somia NV, Schmitt MJ, Vetter DE, Van Antwerp D, Heinemann SF, Verma IM. 1999. LFG: An anti-apoptotic gene that provides protection from Fas-mediated cell death. Proc Natl Acad Sci USA 96:12667–12672.

Stinissen P, Medaer R, Raus J. 1998. Myelin reactive T-cells in the autoimmune pathogenesis of multiple sclerosis. Mult Scler 4:203–211.

Van den Berg A, Maggio E, Diepstra A, de Jong D, van Krieken J, Poppema S. 2002. Germline FAS gene mutation in a case of ALPS and NLP Hodgkin lymphoma. Blood 99:1492–1494.

Watanabe F, Brannan CI, Copeland NG, Jenkins NA, Nagata S. 1992. Lymphoproliferation disorder in mice explained by defects in Fas antigen that mediates apoptosis. Nature 356:314–317.

Weissert R, Kuhle J, De G, Wienhold W, Herrmann MM, Muller C, Forsthuber TG, Wiesmuller KH, Melms A. 2002. High immunogenicity of intracellular myelin oligodendrocyte glycoprotein epitopes. J Immunol 169:548–556.

Yang E, Zha J, Jockel J, Boise LH, Thompson CB, Korsmeyer SJ. 1995. Bad, a heterodimeric partner for Bcl-XL and Bcl-2, displaces Bax and promotes cell death. Cell 80:285–291.

Zang YC, Kozovska MM, Hong J, Li S, Mann S, Killian JM, Rivera VM, Zhang JZ. 1999. Impaired apoptotic deletion of myelin basic protein-reactive T-cells in patients with multiple sclerosis. Eur J Immunol 29:1692–1700.

Zang YC, Hong J, Rivera VM, Killian J, Zhang JZ. 2000. Preferential recognition of TCR hypervariable regions by human anti-idiotypic T-cells induced by T-cell vaccination. J Immunol 164:4011–4017.

Zettl UK, Kuhlmann T, Bruck W. 1998. Bcl-2 expressing T lymphocytes in multiple sclerosis lesions. Neuropathol Appl Neurobiol 24:202–208.

Zhang J, Medaer R, Stinissen P, Hafler D, Raus J. 1993. MHC-restricted depletion of human myelin basic protein-reactive T-cells by T-cell vaccination. Science 261:1451–1454.

Zhong MC, Cohen L, Meshorer A, Kerlero de Rosbo N, Ben-Nun A. 2000. T-cells specific for soluble recombinant oligodendrocyte-specific protein induce severe clinical experimental autoimmune encephalomyelitis in H-2(b) and H-2(s) mice. J Neuroimmunol 105:39–45.

